# Supplementary material for: Translational contributions to tissue specificity in rhythmic and constitutive gene expression
Source: Genome Biol. 2017 Jun 16;18:116. doi: 10.1186/s13059-017-1222-2 (PMC5473967; doi:10.1186/s13059-017-1222-2)
Supplement: Supplementary file 7 — Expression plots for kidney and liver for the 178 common rhythmic genes of Fig. 3c. (ZIP 3338.28 kb) [file 13059_2017_1222_MOESM7_ESM.zip › set_D_shared(178)/Slc16a1_kidney_set_D.pdf]

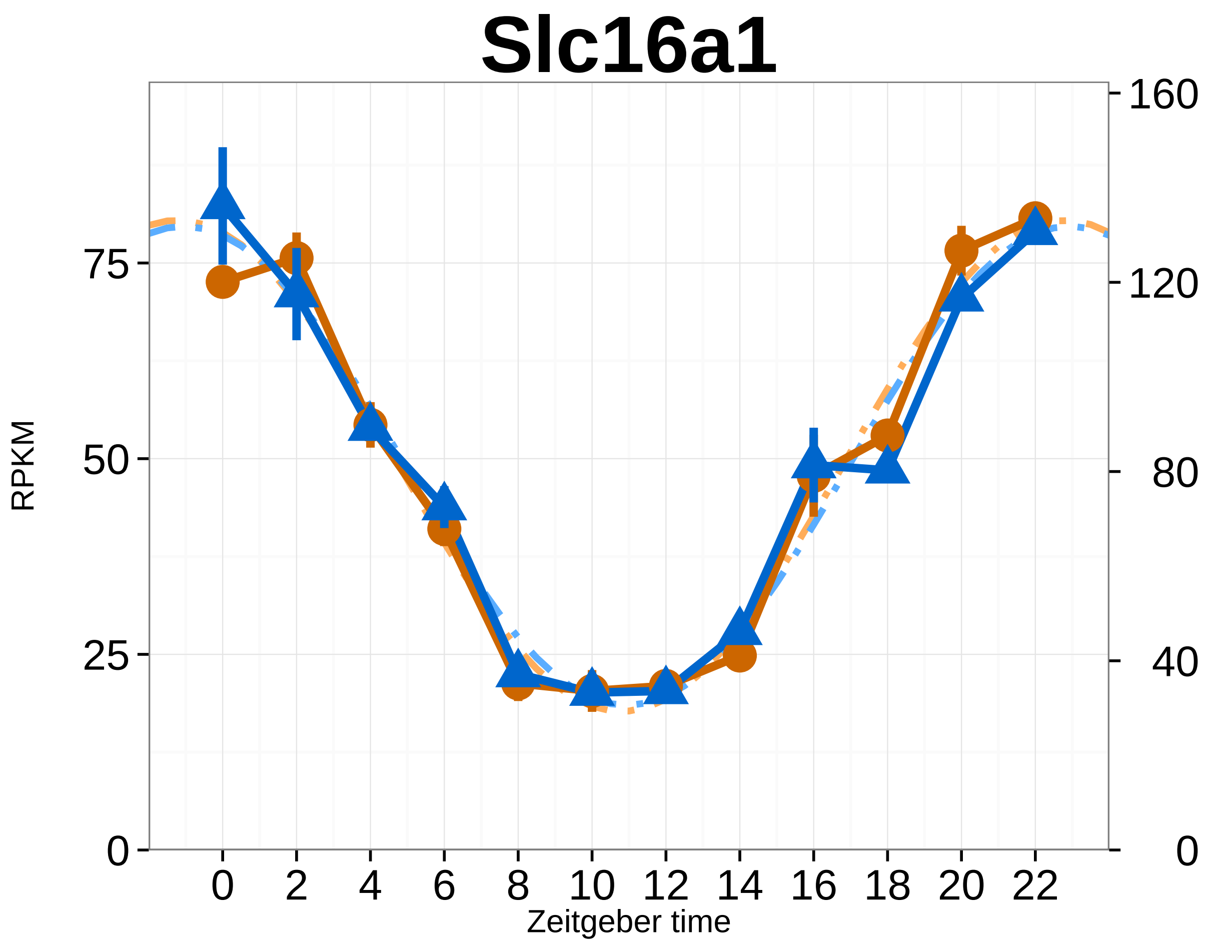

Slc16a1
RPKM
0
25
50
75
0
40
80
120
160
0
2
4
6
8
10
12
14
16
18
20
22
Zeitgeber time

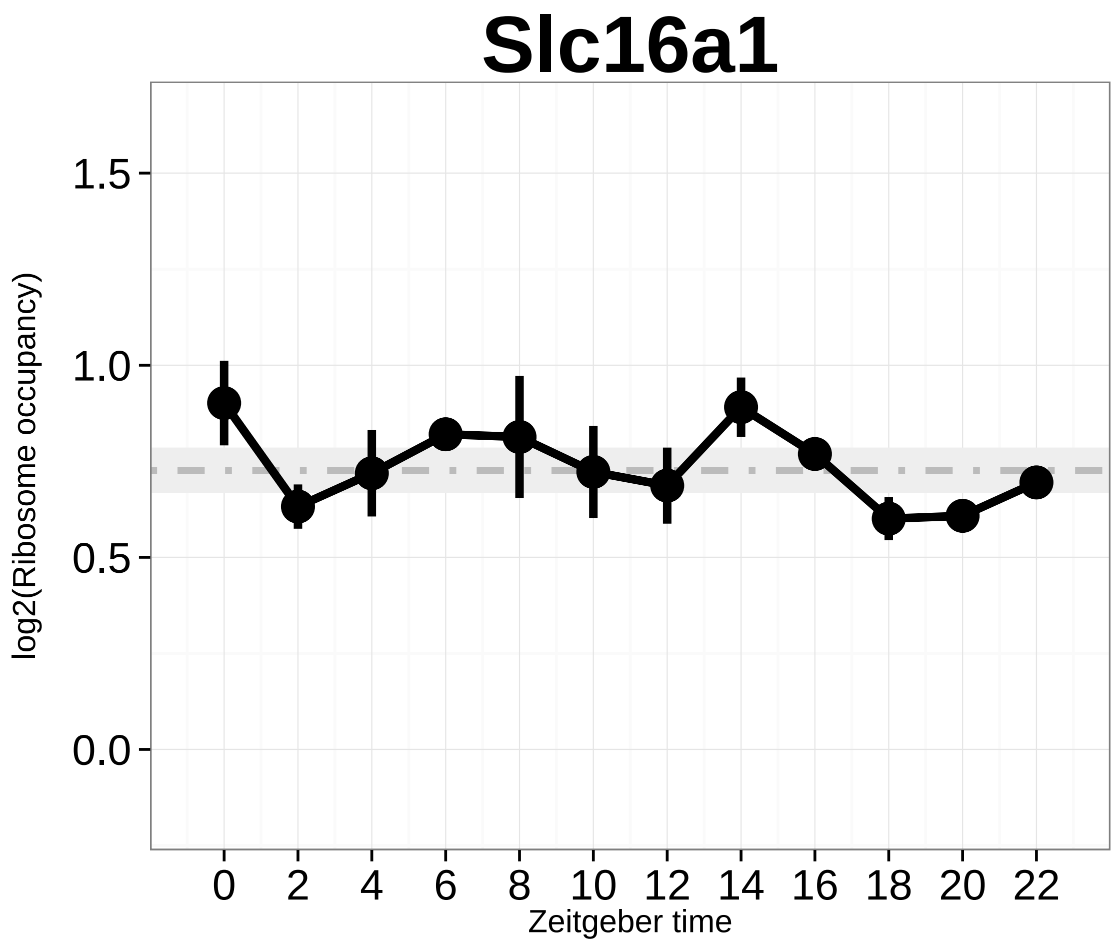

Slc16a1
log2(Ribosome occupancy)
0.0
0.5
1.0
1.5
0
2
4
6
8
10
12
14
16
18
20
22
Zeitgeber time
